# Microglial immunosurveillance after ischemic stroke: dynamic phenotypes, neurovascular interactions, and therapeutic opportunities

**DOI:** 10.3389/fimmu.2026.1893074

**Published:** 2026-08-10

**Authors:** Yingshi Bao, Zixuan Chen, Xianjun Jia

**Affiliations:** 1 Department of Neurology, Suzhou Ninth People’s Hospital, Soochow University, Suzhou, Jiangsu, China; 2 Department of Neurology, The First People’s Hospital of Qidong, Qidong, Jiangsu, China

**Keywords:** blood-brain barrier, immune crosstalk, ischemic stroke, microglia, neuroinflammation, neurovascular unit, stroke recovery

## Abstract

Ischemic stroke induces complex neuroimmune responses that extend beyond acute neuronal injury and involve dynamic interactions among microglia, the neurovascular unit, and peripheral immune cells. Microglia rapidly transition from homeostatic surveillance to damage sensing, cytokine production, phagocytic clearance, and tissue remodeling. Their functions are highly context-dependent: early microglial responses may promote blood–brain barrier protection, debris clearance, and tissue repair, whereas excessive or persistent activation may exacerbate oxidative stress, inflammasome signaling, complement-mediated synaptic pruning, white matter injury, and chronic peri-infarct inflammation. Recent single-cell studies have challenged the traditional M1/M2 framework by identifying multiple transcriptional states that vary across disease stages, brain regions, and systemic immune conditions. Following stroke, microglia interact closely with endothelial cells, pericytes, astrocytes, infiltrating macrophages, neutrophils, lymphocytes, and platelets, thereby linking local neuroinflammation with systemic immunosuppression and infection risk. Therapeutic strategies should therefore seek to redirect specific microglial programs rather than broadly suppress neuroinflammation. Potential approaches include modulating inflammasome and complement signaling, oxidative stress, phagocytosis, lipid metabolism, and repair-associated trophic functions. Clinical translation remains limited by narrow therapeutic windows, patient heterogeneity, difficulty distinguishing resident microglia from infiltrating macrophages, and the absence of validated clinical biomarkers. Future research should integrate longitudinal microglial profiling, neurovascular biology, systemic immune monitoring, and functional recovery measures to develop interventions tailored to specific stages of stroke recovery.

## 1 Introduction

Ischemic stroke remains one of the leading causes of death and disability worldwide, with an estimated 7.6 million new cases added each year (1). Despite advances in endovascular thrombectomy and stroke unit therapy, the time window for reperfusion therapy remains narrow, and most patients do not meet the treatment criteria. Vascular events can trigger a series of cascade reactions, including energy depletion, excitotoxicity, oxidative stress, blood-brain barrier disruption, cell death, and aseptic inflammation (2). These processes do not occur in isolation. Energy failure and calcium overload increase oxidative stress and promote the release of danger-associated molecular patterns, which in turn activate microglia and astrocytes and drive the production of TNF, IL-1β, and IL-6 (3). Barrier injury is also shaped by proteolytic damage. In experimental ischemia, *MMP-9* deletion reduces degradation of blood-brain barrier and white-matter components, supporting a direct link between protease activity and structural disruption of the neurovascular unit (4). However, BBB failure is not a single event with a fixed timeline. Its onset, severity, and underlying mechanism differ across ischemia models, depend on whether reperfusion occurs, and vary according to how barrier permeability is measured (4, 5). Once the barrier is weakened, plasma proteins, complement components, and circulating immune cells gain access to the injured parenchyma, creating a feedback loop between vascular damage and local inflammation. In this setting, microglia occupy a central position because they sense tissue injury, clear cellular debris, remodel synaptic and extracellular structures, and exchange signals with infiltrating leukocytes.

Microglial responses begin within minutes after ischemic onset, but their meaning changes with time and tissue context. A surveillant cell can rapidly become motile, phagocytic, cytokine-producing, and antigen-presenting, while maintaining close interactions with endothelial cells, astrocytes, neurons, and recruited immune cells (6). Early activation may help contain danger signals and support debris recognition; in some settings, microglia-derived TNF can reduce neuronal death through TNFR1 signaling (7). The same response can also become injurious. Inflammasome activation amplifies acute ischemic brain injury (8), and reactivation of the classical complement cascade may contribute to maladaptive synapse elimination (9).

This situational dependence renders the M1/M2 framework insufficient to explain the post-stroke microglial response. Garcia-Bonilla et al. profiled brain and blood cells at 2 and 14 days after MCAO and identified multiple post-ischemic microglial states that became increasingly transcriptionally distinct from monocyte-derived macrophages over time (10). Hammond et al. analyzed 9 specific subgroups throughout life and in injury contexts (11). More recent work has added spatial resolution to this picture. Using single-cell and spatial transcriptomics, Li et al. resolved spatially and transcriptionally distinct microglial subclusters in the acute ischemic brain, including glycolysis-driven ischemic-core-associated microglia and metabolically distinct, myelinotrophic penumbra-associated microglia (12), and Han et al. integrated spatial and single-cell data to map the molecular architecture of the infarct and its border zone (13). Multicolor fate-mapping further showed that post-stroke microglia expand by polyclonal proliferation and that clonality itself contributes to functional heterogeneity, underscoring that microglial diversity cannot be reduced to a single activation axis (14). Therefore, treatment should target the timing, location, and functional status of microglia, rather than broadly inhibiting neuroinflammation. This mini review summarizes the dynamic phenotype of microglia after stroke, their interactions with neurovascular units and peripheral immunity, and the resulting treatment opportunities.

As a narrative mini-review, this article does not follow a formal systematic-review protocol. Literature was identified by searching PubMed and Web of Science up to June 2026 using combinations of the terms “microglia”, “ischemic stroke”, “cerebral ischemia”, “neuroinflammation”, “blood-brain barrier”, “single-cell”, and “neurovascular unit”. We prioritized original experimental studies, including single-cell and fate-mapping work, and landmark mechanistic reports; review articles were cited mainly for conceptual framing. Studies were included when they addressed microglial phenotype, neurovascular or peripheral immune interactions, or therapeutic modulation relevant to ischemic stroke, and were excluded when they concerned unrelated central nervous system disorders without translational relevance to stroke, were limited to conference abstracts, or were not available in English. Given the breadth of the field, citation reflects mechanistic relevance and representativeness rather than exhaustive coverage.

## 2 Dynamic microglial phenotypes after ischemic stroke

### 2.1 Early surveillance, danger sensing, and inflammatory activation

In the healthy brain, microglial processes turn over within hours and collectively survey the parenchyma over approximately one day (15). Ischemia rapidly redirects this surveillance response. ATP and ADP released near damaged cells form chemotactic gradients that are sensed by P2Y12, causing microglial processes to extend toward the lesion (16). HMGB1 provides a parallel danger signal by activating microglial TLR4 after cerebral ischemia-reperfusion (17). As activation progresses, microglia retract their fine processes, enlarge their cell bodies, and acquire inflammatory and phagocytic functions.

The local balance of mediators determines the consequences of this early response. Activated microglia release TNF, IL-1β, IL-6, chemokines, reactive oxygen species, nitric oxide, prostaglandins, and complement proteins. These mediators act on neurons, vascular cells, astrocytes, and recruited leukocytes; IL-1β and TNF promote endothelial activation, whereas complement fragments can label synapses and cellular debris for clearance (18). Inflammasome pathways add another layer of injury. In a rodent stroke model, Denes et al. found that AIM2 and NLRC4, rather than NLRP3, contributed to acute brain injury through ASC-dependent mechanisms (8). Thus, the effect of microglial activation cannot be separated from lesion stage, vascular integrity, or the competing demands of debris removal and tissue preservation.

Timing is one reason microglial pathways have been difficult to target. During the hyperacute phase, resident microglia produce TNF, IL-1β, and IL-6 before substantial neutrophil or monocyte infiltration (19). An intervention that suppresses these mediators early may also interfere with later debris clearance and repair. This shifting balance may help explain the poor clinical translation of broad immunosuppression despite extensive neuroprotective research and numerous unsuccessful clinical trials (20). Responses also differ spatially: the ischemic core, peri-infarct cortex, white matter, hippocampus, and remote regions have distinct oxygen levels, vascular integrity, and neuronal viability. A pathway that is beneficial in one compartment may therefore be harmful in another.

### 2.2 Phagocytosis, repair-associated states, and maladaptive persistence

Phagocytosis is one of the most consequential microglial functions after ischemia. UDP-P2Y6 signaling promotes microglial phagocytosis of injured neurons and cellular debris (21). Efficient clearance can limit secondary inflammation and prepare the tissue for remodeling. Receptors involved in “find-me,” “eat-me,” and lipid-processing pathways determine which targets are engulfed and whether phagocytosis is coupled to inflammatory or reparative downstream programs. Experimental ablation of proliferating microglia exacerbates ischemic injury, indicating that endogenous microglial expansion can contribute to tissue protection after stroke (22).

The effect of phagocytosis depends on whether microglia remove irreversibly damaged material or stressed but still viable neural elements. Neurons that are under stress but still alive can expose phagocytic signals, such as phosphatidylserine or calreticulin, making them vulnerable to phagoptosis. Neher et al. showed that inhibition of the microglial phagocytic mediators MerTK and MFG-E8 reduced delayed neuronal loss after focal ischemia, suggesting that part of the neuronal loss in the peri-infarct region may occur through phagocytosis rather than primary cell death (23). Developmental and neurodegenerative models show that C1q/C3 can tag synapses for elimination (9); whether the same mechanism directly contributes to post-stroke cognitive impairment remains to be established.

Chronically activated and lipid-laden microglia may persist in peri-infarct and remote regions, particularly after large infarcts, aging, diabetes, or recurrent vascular injury. Single-cell analysis by Kim et al. identified *Ifi27l2a* as an aging- and stroke-associated regulator of microglial inflammation; partial reduction of *Ifi27l2a* reduced gliosis, acute and chronic brain injury, and motor deficits in an ischemic stroke model (24). This aging-related inflammatory program overlaps conceptually with the disease-associated microglial signature first described by Keren-Shaul et al., which is characterized by downregulation of homeostatic genes, such as *P2ry12*, *Tmem119*, and *Cx3cr1*, and upregulation of *Apoe*, *Trem2*, *Cst7*, *Lpl*, and *Itgax* (25). Similar features have been detected in aged and stroke-injured brains, together with impaired debris processing, metabolic remodeling, and persistent inflammatory output. The functional consequence of TREM2-associated programs is context-dependent. Keren-Shaul et al. established that TREM2 is required for the full DAM transcriptional program in a neurodegeneration model (25), whereas a recent photothrombotic stroke study found that TREM2 exacerbated ischemic injury through Gpnmb signaling (26). These findings do not support a uniformly reparative or harmful classification.

Host background further modifies these responses. Age, sex, vascular risk factors, prior infection, and microbiome-associated inflammation can all alter microglial state. Wendeln et al. showed that peripheral inflammatory stimulation leaves a long-lasting epigenetic imprint on microglia, producing either trained immunity or tolerance in subsequent neurological disease models (27). The aged brain also accumulates CD8 T cells and develops a stronger inflammatory response to ischemia (28). Such changes affect not only inflammatory intensity but also the metabolic options available to microglia. Early activation is associated with HIF-1α-linked glycolysis, whereas repair-associated states rely more heavily on oxidative phosphorylation, lipid handling, autophagy, and lysosomal function. LXR- and PPAR-γ-regulated lipid efflux pathways may therefore offer targets distinct from conventional cytokine blockade (29) (**Table 1**).

**Table 1:** Key receptors and signalling pathways governing microglial responses after ischemic stroke.

Receptor/pathway	Cellular context	Ligand or upstream signal	Downstream effect on microglia & tissue	Net effect	Ref.
P2Y12	Homeostatic microglia near vessels	ATP/ADP from pannexin-1 and damaged vessels	Directed process extension; closure of BBB gaps within minutes	Protective (acute)	(16, 30)
TLR2/TLR4	Activated microglia in core and peri-infarct	HMGB1, heat-shock proteins, oxidized lipids, matrix fragments	NF-κB–driven TNF, IL-1β, IL-6, ROS; leukocyte recruitment	Mostly damaging if sustained	(17, 18)
NLRP3 inflammasome	Peri-infarct microglia and infiltrating myeloid cells	K^+^ efflux, mtROS, lysosomal rupture downstream of DAMPs	Caspase-1 activation and IL-1β maturation; infarction and edema reduced by MCC950	Damaging	(31)
Complement C1q/C3	Complement-mediated synapse tagging; microglia-astrocyte crosstalk	Apoptotic-cell signals; microglia-derived IL-1α/TNF/C1q	Complement-dependent synapse elimination; induction of A1 neurotoxic astrocytes	Mechanistic evidence; stroke-stage effect unresolved	(9, 32)
MerTK/MFG-E8	Phagocytic microglia in peri-infarct	Phosphatidylserine, calreticulin on stressed neurons	Efferocytosis of debris — but also phagoptosis of viable neurons; blockade reduces delayed neuronal death	Context-dependent	(23)
TREM2	DAM-like/lipid-laden microglia	Lipids, ApoE, anionic phospholipids on damaged cells	TREM2-dependent DAM transition; stroke effects vary by experimental model	Context- and model-dependent	(25, 26)

## 3 Neurovascular interactions and immune crosstalk

### 3.1 Microglia at the neurovascular unit

**Figure 1:**
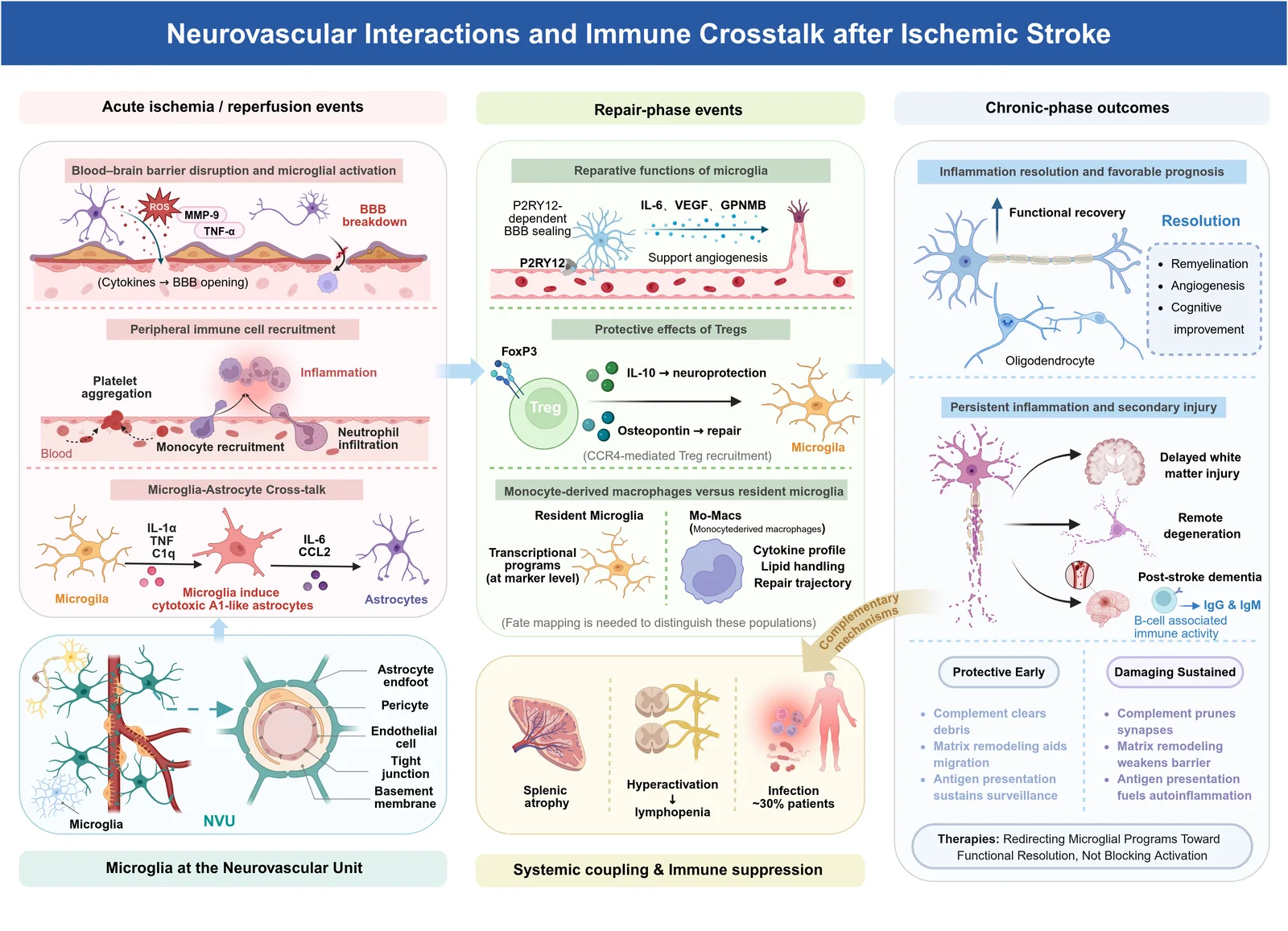
Neurovascular interactions and immune crosstalk after ischemic stroke.

The neurovascular unit comprises endothelial cells, pericytes, astrocytic endfeet, vascular smooth muscle, neurons, basement membrane, and perivascular immune cells. CNS-associated macrophages are also functionally important. In aged mice, Levard et al. found that these cells regulate post-stroke leukocyte trafficking through endothelial adhesion molecules; their loss increased immune-cell entry and worsened neurological dysfunction (33). Microglia influence the same compartment through vascular and glial interactions (33, 34). Lou et al. demonstrated a direct protective mechanism in which P2RY12-dependent microglial processes rapidly close focal BBB openings (30). Conversely, MMP-9-dependent proteolysis can damage BBB and white-matter components after cerebral ischemia (4) (**Figure 1**).

These vascular effects become especially important after reperfusion. Restored flow can rescue threatened tissue, but it also delivers oxygen radicals, plasma proteins, complement, platelets, and leukocytes to an already fragile microvascular bed (35). The ensuing myeloid response is not uniformly destructive. Choi et al. showed that monocyte-derived IL-6 can instruct microglia to support angiogenesis and reconstruct damaged cerebral vessels (36). Microglia also shape astrocyte behavior: IL-1α, TNF, and C1q released by activated microglia induce A1 reactive astrocytes with toxic effects on neurons and oligodendrocytes (32). Astrocytes and microglia therefore participate in reciprocal signaling within the injured neurovascular niche.

### 3.2 Peripheral immune recruitment and systemic coupling

Ischemic injury initiates a peripheral immune response involving multiple leukocyte populations across vascular, perivascular, and parenchymal compartments (18, 37). The Garcia-Bonilla single-cell atlas showed that infiltrating monocyte-derived macrophages overlap with activated microglia at the level of some surface markers, but diverge in cytokine profiles, lipid handling, and repair-associated transcriptional programs; trajectory inference in that study predicted *in situ* trans-differentiation of recruited monocytes into distinct day-2 and day-14 macrophage phenotypes, whereas neutrophils were continuously recruited *de novo* (10). Functional interpretation therefore requires tools that distinguish resident microglia from recruited macrophages, such as fate mapping, parabiosis, or chimeric models (14). Failure to make this distinction has been a recurring source of ambiguity in earlier stroke literature.

Communication between peripheral immune cells and microglia is reciprocal rather than a simple sequence of infiltration. Neutrophils are recruited to the ischemic brain in both experimental models and patients, where their accumulation is associated with inflammatory tissue injury (38). T-cell effects are more stage-dependent. Conventional T cells signal through IFN-γ, IL-17, and GM-CSF, whereas regulatory T cells can restrain acute inflammation through IL-10 (39). During recovery, brain-infiltrating Tregs produce osteopontin, which acts on microglial integrins to promote oligodendrogenesis and white-matter repair (40). Brain Tregs can also release amphiregulin and limit the expansion of neurotoxic astrocytes (41). Enhancing CCR4-dependent recruitment of CD4^+^CD25^+^ Tregs reduced infarct size and improved outcome in a tMCAO model, further implicating microglia-Treg chemokine signaling in repair (42). Platelets add a thromboinflammatory component after reperfusion (43), while sympathetic activation and lymphocyte loss contribute to systemic immunodepression and infection risk (44).

### 3.3 Resolution, remodeling, and secondary injury

Inflammation resolution after stroke requires active cellular and molecular programs rather than passive disappearance of inflammatory signals. In the context of central nervous system remyelination, Miron et al. showed that an M2-like microglial state is required for oligodendrocyte differentiation (45). After ischemic injury, endogenous myeloid responses can support tissue protection, angiogenesis, and white-matter remodeling (22, 46). Zhang et al. recently showed that early-phase microglia attenuation can expand GPNMB-high repair-associated macrophages with pro-angiogenic and pro-remyelinating properties, improving white matter integrity, vascular repair, and long-term recovery after ischemic stroke (46). Consistent with the importance of endogenous myeloid repair programs, ablation of proliferating microglia enlarges the infarct, indicating that microglial proliferation supports tissue protection during recovery (22). When inflammatory resolution remains incomplete, adaptive immune responses may persist during recovery. In a mouse model, Doyle et al. showed that B-cell infiltration contributed to delayed cognitive impairment 4–8 weeks after stroke (47).

Repair-associated pathways can become harmful when they persist beyond their useful phase. Complement aids clearance but can also remove synapses (9), and matrix remodeling facilitates cell migration while weakening barrier structure (4). Prolonged antigen presentation may likewise shift immune surveillance toward a sustained adaptive response (18, 47). These observations favor functional resolution over indiscriminate suppression of microglial activation (23, 48). Biomarkers should distinguish acute injury, repair-associated states, and maladaptive persistence rather than treating inflammation as a single category (10, 48). The same principle applies to outcome assessment: vascular function, network recovery, cognition, mood, and motor performance are more informative than a reduction in inflammatory transcripts alone (47, 48).

## 4 Therapeutic opportunities and translational challenges

Microglia are plausible therapeutic targets because they participate in both acute vascular responses and later recovery. P2RY12-dependent processes help close focal BBB disruptions soon after injury (30), whereas reparative microglial programs can remain active during recovery and improve functional outcome (48). The therapeutic goal is therefore selective modulation rather than global suppression. In a tMCAO model, the NLRP3 inhibitor MCC950 reduced infarction and edema when given 1 or 3 hours after occlusion (31). PPARα-dependent communication between microglia and infiltrating monocytes/macrophages provides another experimentally supported target (49). Recent studies have further identified ZFP384 as a brake on reparative microglial gene expression (48) and TRPC1 SUMOylation as an upstream enhancer of NLRP3 signaling through altered TRPC1-ARRB2 interactions (50).

A central difficulty is that the effect of a pathway changes across the acute, subacute, and chronic phases. Reducing cytokine production early may protect the endothelium, yet prolonged inhibition may impair debris recognition and tissue repair. Phagocytosis presents a similar trade-off: it removes necrotic material but can also eliminate stressed, viable neurons when activated prematurely or excessively (23). Systemic interventions must also account for post-stroke immunodepression and infection susceptibility (44). Attribution is further complicated because many targets are shared by microglia, infiltrating macrophages, astrocytes, endothelial cells, and circulating leukocytes. CSF1R inhibition, for example, directly alters resident microglial viability (51), changing cell abundance as well as activation state.

Biomarkers are needed to connect experimental mechanisms with patient selection. Hybrid TSPO PET/MRI using [18F]FEPPA has shown persistent glial activation six months after ischemic stroke and linked that signal to reduced white-matter microstructural integrity (52). This approach is informative but not microglia-specific, because TSPO is also expressed by astrocytes, endothelial cells, and infiltrating monocytes. Imaging will therefore need to be interpreted alongside circulating immune profiles, BBB measures, extracellular vesicles, and transcriptomic or proteomic data. Clinical variables—including age, reperfusion status, infarct size, infection, and vascular comorbidity—should be incorporated into the same framework. This is consistent with STAIR recommendations to test therapies across age, sex, and comorbidity before clinical translation (53).

Current therapeutic entry points differ substantially in the maturity and specificity of their evidence. Inflammasome signaling (31, 50), complement-dependent responses (9, 32), purinergic pathways (16, 34), CSF1R-dependent microglial survival (51), and PPARα-dependent myeloid crosstalk (49) are all experimentally tractable, but none is confined to a single cell state or disease phase. Treg-based approaches may act more indirectly: increasing brain-infiltrating Tregs with IL-2/IL-2R agonist complexes promoted repair in experimental stroke (40). Future studies should assess functional recovery, delayed degeneration, vascular integrity, cognition, and infection risk rather than relying on infarct volume alone. Delivery also remains unresolved. Systemic treatment is practical but exposes peripheral myeloid cells first, whereas brain-directed strategies require convincing biodistribution and safety data.

## 5 Discussion and future directions

Microglia are active participants in post-stroke injury and repair, not merely markers of inflammation. They sense neuronal and vascular stress, coordinate innate and adaptive immune signals, remove debris, and influence recovery over days to weeks (54). The balance among these functions depends on lesion stage, anatomical compartment, host background, and neurovascular context; no binary marker captures this range. This complexity helps explain the poor translation of broadly suppressive therapies and supports testing interventions in defined cellular states and temporal windows under clinically relevant conditions (53).

Three priorities emerge for the next phase of research. First, longitudinal maps of microglial states should be generated in models that incorporate aging, sex, vascular risk, metabolic disease, and prior infection, because these variables alter both immune responses and treatment effects (28, 53). Second, causal studies must distinguish resident microglia from infiltrating macrophages. Single-cell atlases provide useful reference states, but fate mapping, spatial profiling, and cell-specific perturbation remain necessary for attribution (10, 14). Third, neurovascular injury should be studied as an integrated system in which endothelial activation, barrier failure, vascular macrophage signaling, and leukocyte recruitment determine whether microglial programs support repair or amplify damage (4, 33).

Clinical translation will depend on biomarkers that identify biological stage rather than merely confirm inflammation. Post-acute TSPO PET/MRI findings illustrate the potential to connect glial activation with white-matter injury in patients (52), but no single modality can resolve acute inflammatory injury, debris clearance, vascular remodeling, and repair-associated states. Imaging should therefore be integrated with blood immune profiling and clinical variables such as age, reperfusion status, infarct size, infection risk, and vascular comorbidities. Such biologically stratified designs are consistent with recommendations to test candidate therapies across age, sex, and comorbidity before clinical translation (53).

Recent preclinical evidence that sustaining reparative microglial programs improves recovery (48) supports preserving beneficial, stage-specific functions rather than broadly suppressing microglia. Translation now requires biomarkers that connect these states to vascular stability and patient outcomes (52).
